# Synthetic data as an enabler for machine learning applications in medicine

**DOI:** 10.1016/j.isci.2022.105331

**Published:** 2022-10-13

**Authors:** Jean-Francois Rajotte, Robert Bergen, David L. Buckeridge, Khaled El Emam, Raymond Ng, Elissa Strome

**Affiliations:** 1 Data Science Institute, University of British Columbia, Vancouver, BC, Canada; 2 McGill University and McGill University Health Centre, Montreal, QC, Canada; 3 School of Epidemiology and Public Health, University of Ottawa and Replica Analytics, Ottawa, ON, Canada; 4 CIFAR, Toronto, ON, Canada

**Keywords:** Artificial intelligence, Artificial intelligence applications, Health sciences, Medical science

## Abstract

Synthetic data generation is the process of using machine learning methods to train a model that captures the patterns in a real dataset. Then new or synthetic data can be generated from that trained model. The synthetic data does not have a one-to-one mapping to the original data or to real patients, and therefore has the potential of privacy preserving properties. There is a growing interest in the application of synthetic data across health and life sciences, but to fully realize the benefits, further education, research, and policy innovation is required. This article summarizes the opportunities and challenges of SDG for health data, and provides directions for how this technology can be leveraged to accelerate data access for secondary purposes.

## Graphical abstract

**Figure:**
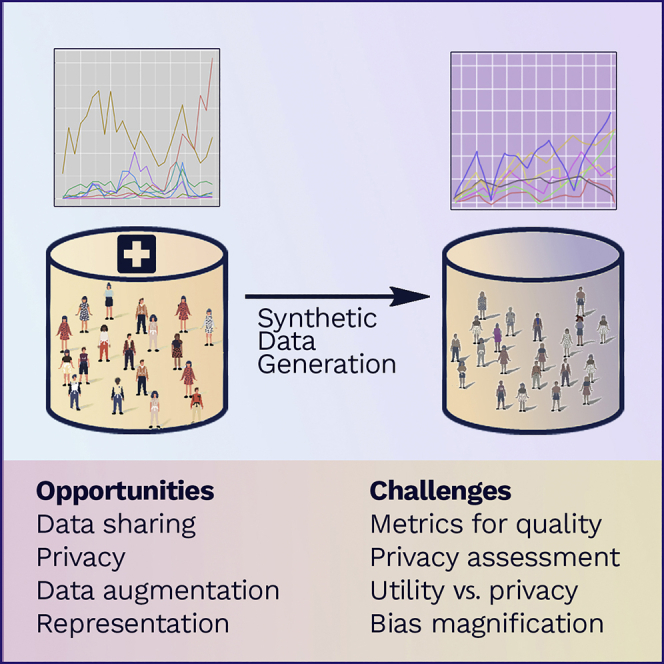

## Introduction

Understanding the opportunity to leverage health data for innovation and care improvement has been a topic of discussion for decades. AI and machine learning (ML) have opened exciting opportunities to harness data within health systems to provide decision support tools to clinicians, develop better treatments, and improve system efficiencies. However, significant barriers to widespread innovation and adoption exist. Because ML applications are data intensive, there is a need to address the challenge of data access.

Privacy concerns are key barriers to health data sharing and data access (van Panhuis et al., 2014), (Kalkman et al., 2019). In the case of published studies, one option is to request datasets directly from their authors, but such data availability is poor (Read et al., 2021). An analysis of the ability to obtain individual level data for research projects from authors of published articles found that the percentage of the time these efforts were successful varied significantly and was generally low (Bauchner et al., 2016) at 58% (Polanin, 2018), 46% (Naudet et al., 2018), 14% (Villain et al., 2015), and 0% (Ventresca et al., 2020). Particularly in EEA countries where the EU General Data Protection Regulation (GDPR) imposes high standards for data sharing that are often difficult to meet in practice (Rabesandratana, 2019), (Bentzen et al., 2021). This raises a particular challenge given that the GDPR is serving as a template regulation around the globe (Bentzen et al., 2021).

Recently, the Public Health Agency of Canada (2022) identified that a “privacy chill”, a slowing or complete restriction on health data sharing, has a significant negative impacts on response to the COVID-19 pandemic, and on Canada’s ability to recruit and retain talented health data scientists who can’t access the data they need to undertake their research (Public Health Agency of Canada, 2021). Technical approaches to enhancing and protecting privacy can help health data stewards overcome “privacy chill”, and share data for secondary purposes. Synthetic data approaches are one such tool.

In November 2021, CIFAR (Canadian Institute for Advanced Research), IVADO (Institute for Data Valorization) and Mila (Montreal Institute for Learning Algorithms) organized a Synthetic Data for Health symposium and workshop to explore the opportunities and challenges of deploying synthetic data approaches across a spectrum of applications in medical research and training, including imaging, genomics, neurophysiology, epidemiology and clinical applications. Synthetic data generation (SDG) is the process of using ML methods to train a model that captures the patterns in a real dataset. Then new, or synthetic, data can be generated from that trained model. The synthetic data, if properly generated, does not have a one-to-one mapping to the original data or to real patients, and therefore has the potential of privacy-preserving properties.

The discussions at the symposium indicated that there is a growing interest in the application of synthetic data across health and life sciences, but that to fully realize the benefits, further education, research, and policy innovation is required. This article summarizes the opportunities and challenges of SDG for health data as raised during the symposium followed by a case study about synthetic PET scans, and provides directions for how this technology can be leveraged to accelerate data access for secondary purposes.

## Opportunities

### Promoting data sharing

In many instances sharing real (i.e., non-synthetic) data for secondary purposes is challenging due to regulatory requirements or ethical concerns which can lead to overly-cautious or protective interpretations, which can lead to delays in dataset sharing or access approvals. Synthetic data could be an attractive alternative. When synthetic data is created with the intent to mimic a given real dataset, it can hold valuable information from the real data such as feature correlations and parameter distributions. Furthermore, it can be used to train statistical models, perform hypothesis-generating studies or simply provide data examples for educational purposes.

In recent years, SDG has made tremendous progress, especially from deep learning generative models. These gains are particularly impressive in the computer vision domain where everyday images can now be generated with strikingly realistic features (Karras et al., 2018), (Dhariwal and Nichol, 2021) and in language generation where realistic text can be written by so-called large language models (Hutson, 2021). Less present in the current news but just as important in medicine are tabular and time series generation with notable applications for electronic health records and biometric measurements (Seyfi et al., 2022). Additionally, related methods also address multi-modal data generation. Sharing synthetic data can help produce more generalizable analyses and facilitate their reproducibility when real data sharing is not feasible.

### Protecting privacy

Although there is no single definition of privacy, the general concept is relatively simple: It defines the level of “protection” against unexpected access to some potentially sensitive information about specific individuals. Patient information is considered highly sensitive and the risks have been traditionally addressed with de-identification methods. However, these methods have proven to be vulnerable to privacy leaks (Sweeney, 2002), (Rocher et al., 2019), (Mandl and Perakslis, 2021). Most synthetic data approaches aim to reproduce populations rather than individuals, with no direct link between individuals in a synthetic sample and individuals in a real sample. While these methods have some challenges as detailed below, if done correctly, synthetic data can be an important tool for data sharing and reducing risks of privacy leaks. A recent model of meaningful identity disclosure risk has shown that synthetic data generated from clinical data can provide 4–5x greater protection against identity disclosure than the real population dataset, falling well below a generally accepted risk threshold (El Emam et al., 2020a). As privacy is always tied to legal issues and how it is (or is not) enforced by laws, we suggest the following introductions of the relationship between synthetic data and the legal landscape, (Bellovin et al., 2018), (El Emam et al., 2020b).

### Data augmentation

Datasets for medical applications are often limited in size because data collection and annotation typically requires the participation of highly trained experts. To address such limitations, data augmentation is a set of techniques to increase the size of a dataset without collecting and annotating more real data. SDG is one such technique and can optimize the statistical information extraction from the real data (Levine et al., 2020), (Nalepa et al., 2019). In its most basic implementation, SDG for data augmentation requires mixing real and synthetic data within the training set of some ML model. For example, the authors of (Levine et al., 2020) have trained a neural network to diagnose different types of ovarian cancer. When synthetic data was added to the training set of real data, the authors claim that the model diagnosis performs as well as a model trained on the real dataset supplemented with more real images.

### Increasing the contribution of underrepresented populations

Small groups of a diverse population may be penalized by ML algorithms in the form of bias. For example, the ubiquitous task of image classification in deep learning results in poor performance when a model is trained on datasets with imbalanced classes (Buda et al., 2018), i.e. one or more classes are significantly underrepresented in the dataset. The errors on the majority class can overwhelm and mask those on the minority classes (Ali et al., 2015). A proposed approach to improve the contribution of an underrepresented group is through data augmentation as mentioned above. The data augmentation of underrepresented groups can lead to improved performance of a model on each subgroup (Rajotte et al., 2021), (Chen et al., 2021). For instance, the authors of Chen et al., (2021) have shown that they can improve the detection accuracy of a rare subtype of renal cell carcinoma by adding synthetic histology images to the training dataset of the detector.

## Challenges

### Assessing quality

Assessing medical synthetic data is an active research topic and many metrics are proposed. Such metrics however, can often be categorized within three qualities: fidelity, diversity and generalization (Alaa et al., 2022).

Fidelity corresponds to the quality of the samples: can they be distinguished from real samples and can valid population inferences be made from the synthetic samples? The validity of such inferences is often referred to as utility and is used as a narrow evaluation of fidelity (Abadi et al., 2016), (Rajotte et al., 2021), (Bergen et al., 2022), (Beaulieu-Jones et al., 2019). More generally, there are two common options for fidelity metrics, computational and human evaluation. Computationally, one can define a distance between the distribution of the real data and the distribution of the synthetic data (e.g. Frechet Inception Distance for images (Heusel et al., 2017) and Hellinger distance for tabular data (El Emam et al., 2022)), or compare statistical model parameter estimates and confidence intervals between the two. It is also a common practice to evaluate the fidelity of the data by asking experts to tell whether a sample is real or synthetic and to report their frequency of success (Salim, 2018), (Beaulieu-Jones et al., 2019), (Choi et al., 2017). Diversity corresponds to the coverage of the real data population: Is a subgroup underrepresented with respect to the original data? Generalization is related to privacy: are the data samples copies of the real data? This question is detailed below, but privacy assessment metrics come in two categories: empirical (through privacy attacks) and formal (from the generation method). These three qualities must be met to a degree determined by the stakeholder and it may be necessary to trade-off one value against another.

### Implementing and assessing privacy

Many ML techniques may lead to a false sense of privacy and SDG is no exception. In general, the privacy of any ML products can be assessed empirically after training with privacy attacks (Murakonda and Shokri, 2020). These attacks can take many forms. Notable ones are data extractions (Carlini et al., 2021), model inversions (Fredrikson et al., 2015) and Membership Inference Attacks (MIA) (Shokri et al., 2017), (Carlini et al., 2022), (Liu et al., 2020). Privacy evaluations of synthetic datasets are usually performed through MIA because of their simplicity and their effectiveness corresponds to an upper bound on privacy. For SDG, MIA receives as input either a synthetic dataset or the model that generated the synthetic data and the attack predicts if a data sample was used for training the SDG model. One must be careful when reporting privacy attack performance metrics, which are often an average-case measure (e.g. accuracy of the membership predictions). As noted and addressed in (Carlini et al., 2022): “If an MIA can reliably violate the privacy of even just a few users in a sensitive dataset, it has succeeded. And conversely, an attack that only unreliably achieves high aggregate attack success rate should not be considered successful”. To address this, the authors propose to use MIA’s true positive rate at low false positive rate as a metric of success, but more generally one could consider creating a metric tailored to the use case.

Another important element to take into consideration is what will be shared about the generation process. On the one hand, it is common in the ML community to share a fully trained SDG model for reproducibility and validation purposes, hence favoring transparency. Furthermore, a fully trained model allows the generation of an unlimited quantity of synthetic data (although the utility of a dataset will not be improved beyond a certain amount of synthetic data added). On the other hand, releasing a fully trained SDG model increases privacy risks. Even releasing an untrained SDG model (i.e. the code) makes the synthetic data more vulnerable because many privacy attacks are based on training “shadow” models which are more effective if they are identical to the actual model used for SDG (Shokri et al., 2017), (Carlini et al., 2022). Since there is a transparency benefit in releasing all the components at the cost of reduced privacy, one has to face a privacy-transparency trade-off.

Federated learning (FL) is often proposed as a privacy preserving alternative for learning algorithms while keeping the sensitive real data local (Rieke et al., 2020). Synthetic data can be created with FL from multiple sites (e.g., hospitals) while keeping the sensitive real data locally. One could conceive a worldwide implementation of such a setting where each site would coordinate with a country, enabling the creation of a synthetic dataset representative of the whole world population or representative of all humans hospitalized or treated for a given condition, such as heart failure or depression. However, FL could also lead to a false sense of privacy if implemented without caution. Indeed, there are many methods demonstrating the privacy vulnerability of exchanging the parameter updates of a model trained on private data, see (Melis et al., 2019), (Zhu et al., 2019) and (Boenisch et al., 2021) for some examples.

The only privacy protection with a predictable degree of privacy for SDG is to include differential privacy (Dwork et al., 2006), (Abadi et al., 2016) (DP) where randomization is added to the learning process to bound the effect of individual patient training records. However, DP in general (i.e. not only applied to SGD) is not a panacea and its implementation is often challenging (Garfinkel et al., 2018) and has received some criticism (Bowen and Garfinkel, 2021). Furthermore, it was shown in a survey (Jordon et al., 2022) that industry players struggle to trust any theoretical privacy claims such as DP without empirical evidence. It is safe to assume that this trust would also need empirical support in medicine.

### Balancing utility and privacy

**Figure 1:**
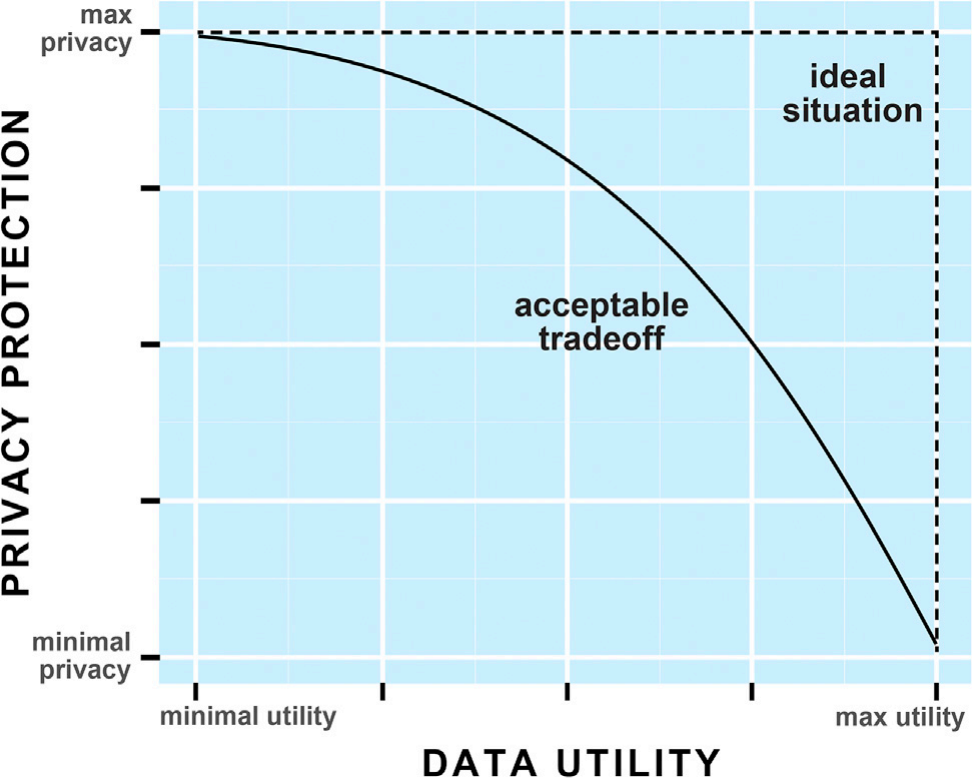
Synthetic data approaches improve the trade-off between privacy and utility A well-crafted synthetic dataset would lie above the acceptable trade-off line as opposed to either the original or de-identified data.

Producing “good” synthetic data often comes at a privacy cost. This is usually referred to as the privacy-utility trade-off and also affects non-synthetic dataanonymization methods. For example, a popular privacy-preserving method, k-anonymization (Sweeney, 2002), reduces data precision such that individuals cannot be singled out. As mentioned above, DP is the only method with formal guaranteed privacy protection, but it often comes at a high and unpredictable utility reduction (Stadler et al., 2022). Moreover, there are examples of DP implementation that could be considered as *privacy-washing*, where the privacy parameters are adjusted for good utility but leading to essentially no privacy protection while benefiting from DP’s reputation of being the best privacy approach (Domingo-Ferrer et al., 2021). There are also other methods for creating synthetic data that demonstrate privacy improvement empirically, e.g., (Mukherjee et al., 2021). Furthermore, the authors of (Stadler et al., 2022) demonstrated empirically that it is challenging to predict what data characteristics will be preserved through well knownSDG methods nor is it possible to anticipate the minimum gain in privacy or utility loss. There is, however, promising work addressing the challenge of controlling both privacy and utility in SDG in a mathematically rigorous way, see (Boedihardjo et al., 2022). Outliers are a particularly clear example of this trade-off because of their fundamental difficulty to be statistically captured based on their uniquely identifying features. If the utility is based on learning from outliers, then a useful and private SDG will be challenging, see (Oprisanu et al., 2022) for a demonstration in SDG of genomic data. Therefore, an ideal private synthetic dataset is created by solving the privacy-utility trade-off (see Figure 1) optimized to the needs of all the stakeholders.

### Avoiding bias magnification from the real data

Most SDG is based on a given real dataset. Real datasets come with their own biases accumulated in the whole data production pipeline: from data collection to data curation (Jo and Gebru, 2020). Synthetic data, like any ML models, will inherit the biases of the data it is based on and potentially magnify them (Torralba and Efros, 2011), (Tommasi et al., 2015). For example, a group underrepresented in the real data might be completely ignored by the SDG process by overgeneralization. Another source of bias is the correlation fallacy, i.e. confusing correlation with causation. Biases should be assessed as much as possible before the release of synthetic dataset, for example by evaluating the quality across subgroups. The evaluation of bias and fairness of a dataset is an active research topic.

## Case study

Our case study demonstrates some of the opportunities and challenges described above focusing on the privacy-utility trade-off. It involves SDG without explicit privacy protection in the training process. Hence, the privacy of the synthetic data is tested empirically with an MIA on the trained SDG model which should not be considered as a privacy guarantee, but an upper bound on privacy as mentioned above. The privacy results are meant as an empirical demonstration of the privacy-utility trade-off.

**Figure 2:**
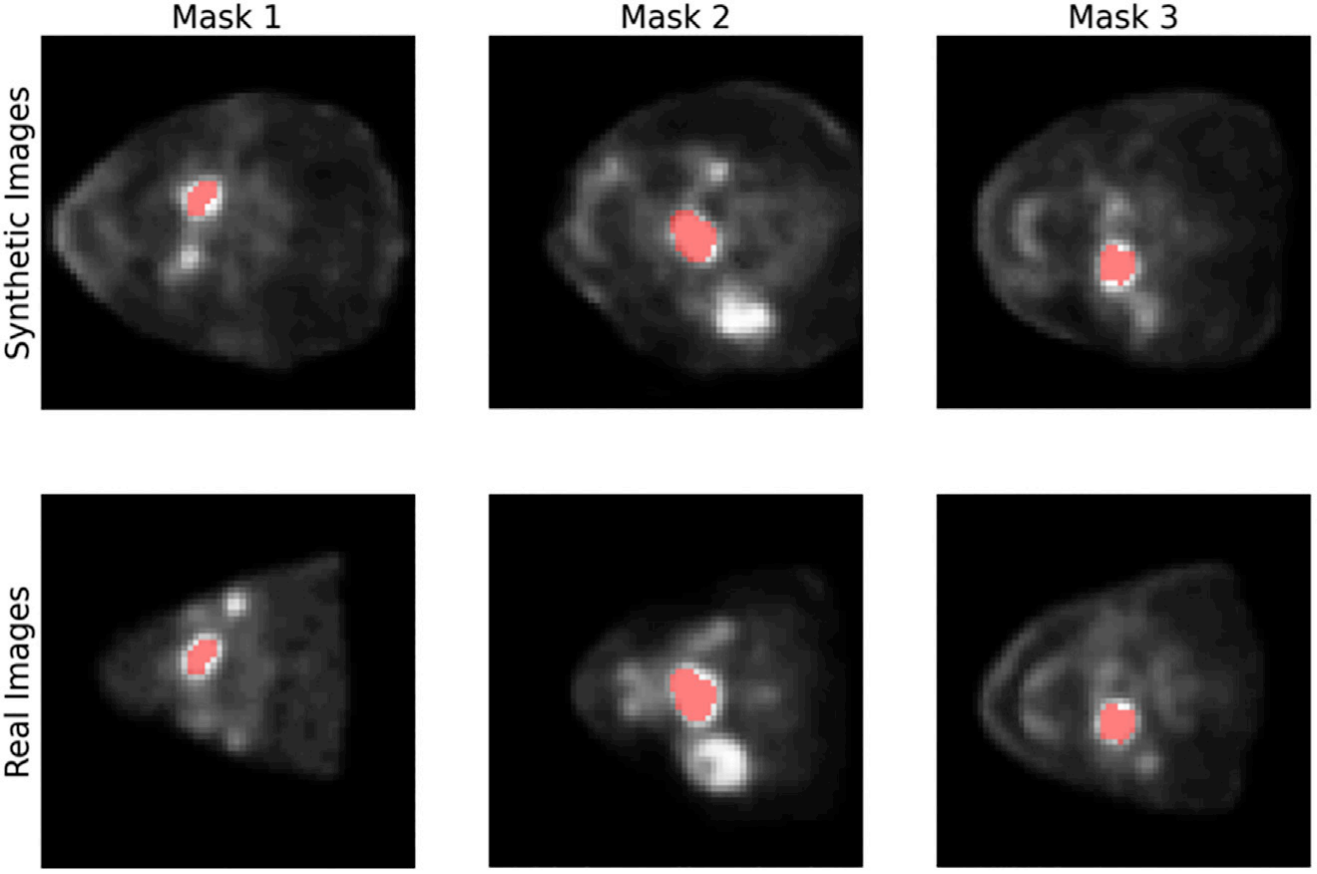
Examples of real and synthetic PET images with tumor masks overlaid in red Each column shows the synthetic image generated by a tumor mask and the real PET image corresponding to the same tumor mask.

The data to be synthesized are 3D head and neck PET images including a tumor mask based on the HECKTOR dataset (Oreiller et al., 2022). The 3D images are composed of 2D transversal slides along the patient’s vertical axis. To create synthetic 3D images, we have used the Transversal GAN model (Bergen et al., 2022), where the 3D PET generation is conditioned by a tumor mask. Figure 2 shows examples of a real and a synthetic slide with corresponding tumor masks overlaid. The purpose of the original dataset is to train a tumor segmentation model. Hence, we define the utility as the performance of a segmentation model trained on the synthetic data. The utility metric is the DICE score, a common performance metric for tumor segmentation.

**Figure 3:**
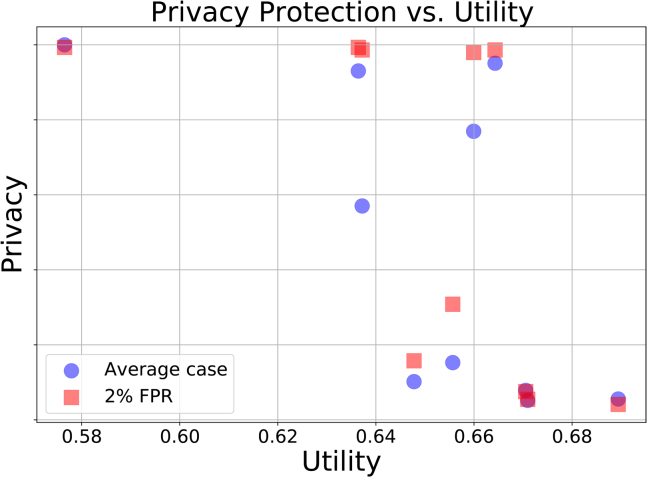
Privacy-Utility values for the generation of 3D synthetic PET images Each value corresponds to a different amount of training iteration of the SDG model. The blue circles correspond to the average case privacy as described in the text and the red squares correspond to privacy at low (2%) false positive rate MIA. The privacy values have been resized to fit on the same axis.

We use two definitions of privacy which were discussed above, one as an average case of the MIA success and the other at a low false positive rate (2%).The average case privacy is determined from the re-identification accuracy of an MIA attack on the SDG model. The low false positive rate privacy is determined from the true positive rate of re-identification. Based on these definitions, we have produced the privacy-utility trade-off curve shown in Figure 3. Each value on this plot corresponds to a different number of training iterations of our SDG model. It is well known that the more training iterations on a dataset, the better the model is until a certain point where it “overfits” the training data. This overfitting is a major cause of privacy leakage. This privacy-utility plot can be used to decide at which iteration a model has been trained enough to satisfy both privacy and utility requirements. In our case study, the DICE score of 0.66, before the privacy steep drop, is to be compared with the 0.7 DICE score of a model trained on the real data.

This case study shows how the number of training iterations could be used to find an optimal model to create a synthetic dataset within acceptable (empirical) privacy and utility bounds. These results however, do not cover all the elements that evaluation needed for a synthetic dataset. Indeed, beyond the privacy and utility metrics mentioned above, further evaluation should be performed to make sure that any subgroups are not over penalized. A reduced utility for a given subgroup could originate from a diversity limitation often affecting GAN-based SDG which is known as mode collapse, a failure case where the synthetic data contains less variety than the original data, see (Han et al., 2018) for an example in a medical SDG. The transversal GAN paper (Bergen et al., 2022) explores this issue by comparing the distributions of multiple radiomic features calculated over the segmentations of the real and synthetic tumors. In addition, if any further downstream tasks such as classification or clustering are relevant to the synthetic data, it is worth defining utility metrics and including them in the evaluation.

## Future directions and recommendations

There are many important applications of SDG to health data, including for training and education in clinical data sciences (James et al., 2021), and the development and testing of new ML-based clinical decision-support tools. Synthetic data approaches are an important set of tools to help protect patient privacy, augment small datasets, and reduce bias against subgroups.

Reflecting on synthetic health data opportunities and challenges is timely in the current state of our data-driven world. In September 2021, the UN High Commissioner for Human Rights called for urgent action regarding artificial intelligence risks to privacy, stating that “… filling the immense accountability gap in how data is collected, stored, shared and used is one of the most urgent human rights questions we face” (OHCHR, 2020). Updating privacy legislation and the development of data and AI regulations are top priorities for many jurisdictions from the EU ((European Union, 2021)), to the US (Lander, 2021) and Canada ((Canada, Office of the Privacy Commissioner, 2021), (Quebec, National Assembly, 2021), (Ontario, Ministry of Government and Consumer Services, 2021)). New technological approaches to protecting privacy like synthetic data will also require policy and innovation, and clinicians and scientists working in ML for health applications need to engage with policymakers to ensure they understand both the opportunities and challenges that synthetic data presents for protecting health data privacy. It is important to define best practices and standards for SDG in collaboration with regulators, privacy officers, and research ethics boards.

At the same time, we have seen first-hand how the lack of data access and sharing has hampered our ability to develop real-time monitoring, modeling, and a coordinated public health response to the COVID-19 pandemic by jurisdictions across the world. The impact of the “privacy chill” described by the pan-Canadian Health Data Strategy’s Expert Advisory Group on our ongoing public health crisis has contributed to the human toll of the pandemic. Beyond local hospitals or even one country doing SDG on their own, there are various collaborative settings such as (1) multiple countries sending their data to a trusted third party; and (2) collaborators without a trusted central node having real data exchanged hands.

Given the opportunities that data science and ML provide to leverage data within our health systems to develop new treatments, deliver better care and reduce costs, there is a strong case for investing in further research and development of synthetic data approaches. For those approaches to translate into real-world applications will require extensive discussion, debate and understanding by all those concerned with preserving health data privacy, from scientists to innovators, from peer and ethics review committees, to hospital administrators and data stewards, to privacy commissioners and policymakers. Given everything we have learned about the lost opportunities with poor data access over the last two years, there is an urgent need to develop and adopt privacy-enhancing technologies to enable data sharing. Regulations governing the sharing and reuse of data are only getting stricter and the role of techniques such as SDG will need to become more prominent. An acceleration in developing frameworks for evaluating the utility and privacy of synthetic data would be a good starting point as that would make it easier to improve SDG methods, and for data custodians to decide how and when to use them.
